# Augmented memory: a survey of the approaches to remembering more

**DOI:** 10.3389/fnsys.2014.00030

**Published:** 2014-03-03

**Authors:** Christopher R. Madan

**Affiliations:** Department of Psychology, University of AlbertaEdmonton, AB, Canada

**Keywords:** memory, cognitive enhancement, neuroethics, transcranial stimulation, nootropics, mnemonics

Given that our ability to remember is inherently limited, one “solution” is to artificially enhance memory. Here I discuss four general approaches that have been developed to augment human long-term memory: nootropics agents, brain stimulation, mnemonic strategies, and external aids. The former two have only been recently developed in the field of systems neuroscience, and have become the focus of ethical debate. For example, some ethicists question the propriety of artificial memory enhancement in healthy individuals. As I demonstrate here, all four methods have been considered ethically suspect at one time or another. In medieval times, the use of mnemonics was considered immoral by many, and even the use of written texts as memory aids has been suggested as producing the appearance of knowledge, void of actual knowledge. Here I present a summary of each approach, beginning with those that fall within the scope of systems neuroscience, and discuss considerations critical to each of their respective ethical debates.

## Nootropics

Nootropics are pharmacological agents consumed solely for the purpose of cognitive enhancement, sometimes referred to as “cosmetic use.” Most nootropics are prescription drugs developed to treat a disorder, but are instead taken off-label for cognitive enhancement. However, nootropics by the broadest definition can also include well-accepted psychoactive compounds including caffeinated drinks and energy drinks. Currently there is some evidence that caffeine can enhance memory (Jarvis, 1993; Hameleers et al., 2000; Borota et al., 2014), however, results are not conclusive (Nehlig, 2010). Active ingredients used in energy drinks, such as taurine and guaraná, can also enhance memory (Alford et al., 2001; Haskell et al., 2007). There is also evidence that nicotine and sage have beneficial effects on memory (Tildsley et al., 2005; Heishman et al., 2010). In general, nootropics can enhance memory encoding, but also may influence retrieval processes.

Numerous drugs are taken off-label for their nootropic properties (see Lannii et al., 2008, for a review). Piracetam is credited as the first nootropic (Winblad, 2005; Winnicka et al., 2005; Margineau, 2011) and has demonstrated memory enhancing effects (Dimond and Brouwers, 1976). Unlike most drugs, piracetam has a very weak affinity to receptors (Winblad, 2005; Margineau, 2011) and its mechanism of action is unclear. Since the initial report of piracetam's memory facilitation in 1976, pharmacology research has attempted to identify other compounds with memory enhancing abilities. Modafinil, marketed as a treatment for sleep disorders, has been found to enhance memory (see Repantis et al., 2010, for a review). Of particular interest, Kohli et al. (2009) found modafinil to enhance both quality and speed of memory. Additionally, memory enhancements were sustained after continued administration. Recent research with ampakines in non-human primates have also yielded promising results (e.g., Porrino et al., 2005). While many other nootropics also exist, such as adderall and ritalin, these drugs do not enhance memory directly, but can effect other cognitive abilities (de Jongh et al., 2008; Lannii et al., 2008).

Recent studies have shown that university students around the world are taking nootropics to improve academic performance (e.g., Eickenhorst et al., 2012; Dietz et al., 2013; Kudlow et al., 2013; Mazanov et al., 2013; Partridge et al., 2013; Sattler and Wiegel, 2013), though prevalence rates vary greatly between studies. Eickenhorst et al. (2012) surveyed students to determine the motivations for using nootropics and found that improving concentration, vigilance, and cognitive potential ranked the highest, though enhancing memory was also a major motive. However, it can be argued that nootropics lead to an uneven playing field, where wealthier individuals, who have access to nootropics, can perform better academically. While the ethics of nootropics is an emerging topic, the consumption of drugs to enhance performance is a time-worn topic within the field of athletics, where such drugs are considered cheating. Additionally, it is unclear what would constitute enhancement versus therapy—consider an older adult with gradually decreasing memory, is it “fair” to use nootropics to perform at the same level as a young adult, or would this be cheating?

## Brain stimulation

Brain regions can be non-invasively stimulated using transcranial magnetic stimulation (TMS) or transcranial direct current stimulation (tDCS). Briefly, both of these techniques modulate the excitability of neurons in the targeted regions. See Sparing and Mottaghy (2008) for a technical review of TMS and tDCS methodology. As both of these methods have limited depth of penetration, the main memory-related regions (i.e., the medial temporal lobe) cannot be targeted. However, the dorsolateral prefrontal cortex (DLPFC) has been shown to be important to memory encoding and is often the target of TMS or tDCS in memory studies (e.g., Marshall et al., 2004; Gagnon et al., 2011; Javadi and Walsh, 2012; Javadi et al., 2012). As both of these methods are unlikely to globally enhance cognitive function, but instead increase activity in one region while decreasing activity in another (net zero-sum model; Brem et al., 2014), it is important to consider the role of DLPFC in memory. The DLPFC is often associated with attention and working-memory (e.g., Lebedev et al., 2004). Though the aforementioned studies focused on DLPFC stimulation, other regions of the PFC have also been related to memory function (Blumenfeld and Ranganath, 2007). The PFC in general has been associated with several facets of higher-level cognition (see Wood and Grafman, 2003, for a review), with an emphasis on goal planning (Passingham and Wise, 2012). One view of the relation between attention and episodic memory is that information must first be attended to before it can be successfully encoded into memory. Along with this, working memory can serve as an intermediate process between attending to the information and the encoding of it.

To stimulate a brain region using TMS, pulses need to be applied concurrent with the memory task (also see Walsh and Cowey, 2000). As a result, TMS can only be effectively used within a controlled (i.e., laboratory) setting and cannot be readily used as a memory enhancement technique by one's self. In contrast, though not done in conjunction with a memory task, changes in cortical excitability due to tDCS stimulation have been shown to persist 90 minutes after stimulation (Nitsche et al., 2003, 2005), and in some cases can have persisting after-effects even 30 days later (Boggio et al., 2008). Additionally, tDCS devices are becoming available to the public (Nature Editorial, 2013), targeted at improving attention and reaction time in gamers. Of particular concern, this also allows parents to use tDCS in-home with their children to hasten learning (Kadosh et al., 2012), despite the effects of tDCS on development being unclear. Kadosh et al. (2012) suggest that using tDCS to enhance learning may be viewed as cheating as it can confer an “unearned,” and thus unfair, advantage to the user. However, hiring a tutor could be similarly unfair as the tutor's guidance would make learning easier.

Memory can also be enhanced through invasive stimulation. Of course, invasive methods cannot be ethically conducted on the same scale and with the same control measures as with non-invasive methods. Hamani et al. (2008) describe a case where a patient was implanted with a deep brain stimulation (DBS) device targeted at the hypothalamus to treat morbid obesity. Through post-operative CT scans, the researchers estimated that the electrodes were located in the hypothalamus, but two were notably proximal to the fornix. Initial stimulation of one electrode evoked an autobiographical memory from decades prior. Of particular relevance, the patient developed enhanced memory function. Hamani et al. found that DBS led to greater activation in the patient's hippocampus and parahippocampal gyrus. Suthana et al. (2012) implanted DBS electrodes in the entorhinal cortex of epilepsy patients and found enhanced spatial memory. Generally, such DBS studies are only conducted with patients that have already been implanted with electrodes for non-memory reasons (e.g., localizing epilepsy foci), but recent successes may soon lead DBS to be used as a treatment for patients with memory impairments. Laxton et al. (2010) implanted DBS electrodes in the fornix in Alzheimer's patients. Stimulation drove activity in entorhinal and hippocampal regions and improved memory.

**Figure 1:**
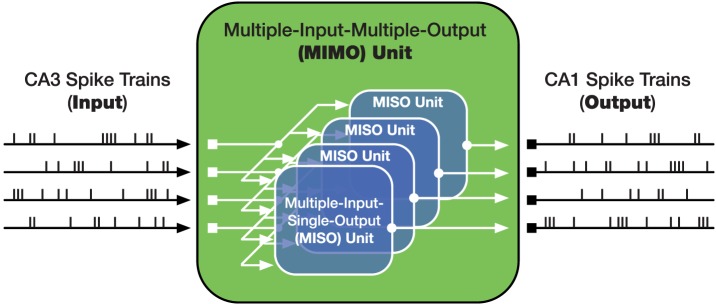
**Graphical representation of the mathematical model developed by Berger et al. (2011) to simulate connectivity between CA3 and CA1 of the hippocampus**. Figure adapted from Berger et al. (2013).

Recent research in non-human primates has also lead to the development of a neuroprosthetic device that enhances memory through task-specific activity (Hampson et al., 2013). In contrast to DBS, where fixed frequency stimulation is used to activate regions, this neuroprosthetic device is built using a nonlinear systems approach that computes multiple-input-multiple-output (MIMO) associations with CA3 spike trains as inputs and CA1 spike trains as outputs (see Figure 1; Berger et al., 2011, 2013). More recent developments with this model have allowed for the transference of memories between individuals (Deadwyler et al., 2013). Converging with non-invasive methods, the MIMO device has also been implanted in the PFC and shown to enhance memory (Hampson et al., 2012).

Invasive stimulation techniques involve a myriad of additional ethical issues that are not present with non-invasive methods. For instance, it is unethical to implant stimulation devices purely for research, an upcoming ethical issue will be the option to opt for elective brain implants. As people are already able to opt for cosmetic surgery in the absence of any medical issues, it seems reasonable that one should also be able to elect for cognitive enhancements without a medical need. Along these lines, individuals who get cosmetic surgery can still compete in a beauty pageant without being considered “cheaters.” Simply put, assuming no significant risks, should an operation to improve attractiveness be more ethical than improving cognition? That being said, further research is needed before one can ask their family physician for a referral to get a “memory implant.”

## Mnemonics

The least controversial approach to enhancing one's memory is to use a strategy (i.e., mnemonics), sometimes referred to as internal aids. Countless strategies exist to improve memory encoding, several of which can be used spontaneously, such as rote repetition, making a sentence or story, imagining the to-be-remembered words, and forming a mnemonic using the first letters of the words (Harris, 1980; Intons-Peterson and Fournier, 1986). Additionally, everyday memory experts such as waiters (Ericsson and Polson, 1988; Bekinschtein et al., 2008), taxi drivers (Maguire et al., 2000), and chess masters (Chase and Simon, 1973; Gobet and Simon, 1996) use more specialized strategies.

For more generalizable strategies it is most useful to focus on individuals who have trained themselves to have superior memory. Maguire et al. (2003) compared superior memorizers, those who placed highly in the World Memory Championships, to controls. Most superior memorizers reported using the method of loci, a strategy first developed by ancient Greeks, and sometimes referred to as a “memory palace.” In this strategy, one imagines a familiar environment (usually their home) and walks through this imagined environment, placing the to-be-remembered items at various locations (loci). To recall the items, the individual imagines walking through the environment and sees the items once again (also see Yates, 1966; Raz et al., 2009; Legge et al., 2012; Madan and Singhal, 2012). Importantly, superior memorizers have been found to exhibit differences in functional activations in the hippocampus and retrosplenial cortex (Maguire et al., 2003). Other techniques can also be used to achieve extraordinary memory, such as chunking, where information is hierarchically grouped (e.g., Chase and Simon, 1973). The primary flaw of mnemonics is that effective use often requires extensive practice.

Considering the ethics of mnemonic use, some Christians in the middle ages viewed mnemonics as immoral, considering them to be magic, in part due to their pagan roots (Yates, 1964, 1966). However, others embraced it and used it as a tool for the remembrance of Biblical text.

## External aids

External aids such as written lists can artificially improve memory (see Harris, 1980, and Intons-Peterson and Fournier, 1986, for a comprehensive list of aids), and are primarily used as retrieval cues. Modern technology has vastly increased the capacity and convenience of external aids, particularly due to the advent of cell phones (Wilson et al., 1999; Wade and Troy, 2001; Svoboda et al., 2012). While the use of external aids is relatively innocuous, it is not free of debate. In *Phaedrus*, Plato (360 BC, 275a) recounts a conversation where Socrates cautions Phaedrus against being too dependent on written texts:

Trust in writing will make them remember things by relying on marks made by others, from outside themselves, not on their own inner resources, and so writing will make the things they have learnt disappear from their minds. [Writing] is a potion for jogging the memory, not for remembering. You provide your students with the appearance of intelligence, not real intelligence. Because your students will be widely read, though without any contact with a teacher, they will seem to be men of wide knowledge, when they will usually be ignorant.

This passage still rings true and may even be more relevant today. With ready access to the Internet, people have even less reason to remember information directly, instead remembering where to find the information, but not the information itself (Sparrow et al., 2011). However, information stored using external aids is less susceptible to memory biases (e.g., false memories, primacy and recency effects). Future neuroimaging research comparing cued versus uncued memories using external aids may provide additional insight into the neuronal mechanisms of memory.

## Conclusion

Recent advances in systems neuroscience have provided new approaches to artificially enhancing memory; however, these have not come without controversy. While it is not possible to resolve these debates without further discussion, it is important to acknowledge that although other approaches to artificially enhancing memory appear innocuous now, this has not always been the case. One direction forward is to draw parallels with other fields that have observed similar debates in the past, such as in the case of performance-enhancing drugs for athletes and cosmetic surgery for beauty competitions, and benefit from the discourse that has already surrounded their own ethical disputes.

### Memory augmentation as an intervention

While the focus of this article is the use of augmentation to enhance memory in healthy individuals, it is important to acknowledge that these methods should be equally, if not more, beneficial to individuals with diminished memory function (e.g., older adults and Alzheimer's patients). Additionally, it is possible that diminished function can be a form of enhancement (Earp et al., 2014). With respect to impaired memory as an intervention, one such case would be patients with post-traumatic stress disorder.
